# Cell Proliferation (KI-67) Expression Is Associated with Poorer Prognosis in Nigerian Compared to British Breast Cancer Women

**DOI:** 10.1155/2013/675051

**Published:** 2013-04-11

**Authors:** Ayodeji O. J. Agboola, Adekumbiola A. F. Banjo, Charles C. Anunobi, Babatunde Salami, Mopelola Deji Agboola, Adewale A. Musa, Christopher C. Nolan, Emad A. Rakha, Ian O. Ellis, Andrew R. Green

**Affiliations:** ^1^Department of Morbid Anatomy and Histopathology, Olabisi Onabanjo University, Sagamu, Nigeria; ^2^Specialist Laboratory, Idi-Araba, Lagos, Nigeria; ^3^Department of Surgery, Olabisi Onabanjo University, Sagamu, Nigeria; ^4^Department of Medical Microbiology and Parasitology, Olabisi Onabanjo University, Sagamu, Nigeria; ^5^School of Molecular Medical Science, Oncology Unit, University of Nottingham, Nottingham, UK

## Abstract

*Background.* Black women with breast cancer (BC) in Nigeria have higher mortality rate compared with British women. This study investigated prognostic features of cell proliferation biomarker (Ki-67) in Nigerian breast cancer women. *Materials and Methods.* The protein expression of Ki-67 was investigated in series of 308 Nigerian women, prepared as a tissue microarray (TMA), using immunohistochemistry. Clinic-pathological parameters, biomarkers, and patient outcome of tumours expressing Ki-67 in Nigerian women were correlated with UK grade-matched series. *Results.* A significantly larger proportion of breast tumours from Nigerian women showed high Ki-67 expression. Those tumours were significantly correlated with negative expression of the steroid hormone receptors (ER and PgR), p21, p27, E-cadherin, BRCA-1, and Bcl-2 (all *P* < 0.001), but positively associated with EGFR (*P* = 0.003), p53, basal cytokeratins: CK56, CK14, triple negative, and basal phenotype using Nielsen's classification (all *P* < 0.001) compared to UK women. Multivariate analyses showed that race was also associated with BCSS independent of tumour size, lymph node status, and ER status. *Conclusion.* Ki-67 expression was observed to have contributed to the difference in the BCSS in Nigerian compared with British BC women. Therefore, targeting Ki-67 in the indigenous black women with BC might improve the patient outcome in the black women with BC.

## 1. Introduction 

There are discrepancies in mortality rates among the nationalities with Caucasian women having a low mortality rate compared with black women [1–6]. In African-American women, the effect is more pronounced in younger women compared with European-American women [3, 7]. Although there are international variations in the mortality rates of breast cancer, the explanations that predispose the cells to aggressive tumour phenotype and patient survival are not clear.

Cell proliferation is one of the important driving steps in patients with aggressive tumour phenotype and patient survival [8, 9]. Cell proliferation is controlled by regulatory proteins that ensure orderly progression of the cells through the check points of the cell cycle [10–12]. The cell cycle checkpoints comprise of 4 important phases which are arranged sequentially: G1 phases that prepare their machinery for duplication, S phase is responsible for the genomic materials' duplication, G2 phase is known as intervention phase, and the M phase that controls mitosis [13]. Abnormal cell cycle regulatory protein activities are central to increase cell proliferation, poor maintenance of chromosomal integrity, and therefore encouraging tumour development [11, 12].

KI-67 is one of the proliferative markers strongly linked to cell cycle control. During mitosis, this protein is tightly regulated by protease upon completion of its activity, such that its half life is less than 90 minutes [14, 15]. It is expressed in all proliferative cells both in normal and malignant cells. In addition, it represents easy and reliable methods of assessing the cell cycle pathways particularly in breast cancer [14, 15]. The prognostic significance of this protein in BC of western women has been reported [8, 9, 16, 17]. There is a positive correlation between KI-67 protein expression, cell proliferation rate, and the active phase of the cell cycle in invasive breast carcinoma. High KI-67 is associated with tumours of high grade, large size, and lymph node involvement, basal phenotype, and ER and PR negative and HER-2 positive tumours [18, 19]. KI-67 is also highly expressed among breast tumours from African-American women compared with Caucasian women, most especially among younger women [20, 21].

Although few studies have been reported on prognostic significance of KI-67 on African-American women with breast cancer as one of the reasons for increased likelihood of African-American women presenting with advance stage at diagnosis, these findings might not completely explain the roles of KI-67 in the indigenous African women with BC, because of the differences in the environmental factors. Up until now, the tumour biology that may contribute to the differences observed in the ethnic nationalities is not completely understood especially on the indigenous black women with breast cancer [22, 23]. We therefore, hypotheses that alteration of KI-67 expression may contribute to the tumour biology observed among the ethnic nationalities with BC.

Thus, the aim of this study is to investigate the KI-67 expression using immunohistochemistry in breast tumours from Nigerian women and to compare them to a well-characterised series of BC from Caucasian women living in the UK, in order to establish whether the differences between the two nationalities are due to KI-67 tumour biology.

## 2. Material and Methods

### 2.1. Patients

The Nigerian patient cohort comprised formalin-fixed paraffin embedded (FFPE) breast cases from 308 women presenting at the Olabisi Onabanjo University Teaching Hospital, Sagamu, and Histopathology Specialist laboratory, Idi-Araba, Lagos, Nigerian, from January 2002 to December 2008. Clinical history and tumour characteristics including age, menopausal status, tumour type, histological grade, tumour size, lymph node status, and vascular invasion were assessed in a standardised manner for all the patients.

The tissue sections were reevaluated for histological features such as tumour grade and type. Patient outcome and treatment data were retrieved from the patient's records. All patients were treated with combination of classical chemotherapy cyclophosphamide, methotrexate 5FU, and hormonal therapy (tamoxifen). Eighty-five out of the patients (42.5%) received radiotherapy. Patients were followed up for at least 60 months (260 weeks).

A UK patient cohort of 1,902 primary operable invasive breast carcinoma cases was from the well-characterised Nottingham-Tenovus Primary Breast Carcinoma Series consisting of women presenting between 1986 and 1998. All patients were assessed in a standardised manner for clinical history and tumour characteristics [24–27]. Patient management was based on tumour characteristics by the Nottingham Prognostic Index (NPI) and hormone receptor status. Patients with an NPI score ≤3.4 received no adjuvant therapy, and those with a NPI score >3.4 received Tamoxifen if estrogen receptor (ER) positive (±Zoladex if premenopausal) or classical cyclophosphamide, methotrexate, and 5-fluorouracil if ER negative and fit enough to tolerate chemotherapy [28]. Survival data including breast cancer specific survival (BCSS) and disease-free interval (DFI) were maintained on a prospective basis. DFI was defined as the interval (in months) from the date of the primary surgical treatment to the first locoregional (including invasive malignancy and ductal carcinoma in situ) or distant recurrence. BCSS was taken as the time (in months) from the date of the primary surgical treatment to the time of death from breast cancer.

Of this series, 841 cases had complete data on the following immunohistochemical markers: estrogene receptor (ER), progesterone receptor (PgR), cytokeratins (CK5/6, CK14,) EGFR (HER1), HER2, BRCA1, placental-cadherin (P_-cadherin), Epithelial-cadherin (E-cadherin), p53, p21, Bcl-2, MDM2, and MDM4 [24–27]. In order to compare the Nigerian and UK series with respect to biomarkers and patient outcome, a grade-matched UK control groups to the Nigerian cohort, comprising of 308 patients, was generated from the previous patients. Within the UK tumour grade-matched cohort, 202 were selected for patient outcome 122/202 (60.4%), 53/202 (26.2%) had died at 60 months and 80/202 (39.6%), 149/202 (73.8) remained alive in Nigeria and UK respectively.

The Reporting Recommendations for Tumour Marker Prognostic Studies (REMARK) criteria, recommended by McShane et al. [29], were followed. This study was approved by the Medical Advisory Committee, Olabisi Onabanjo University Teaching Hospital and by the Nottingham Research Ethics Committee 2 under the title of “Development of a molecular genetics classification of breast cancer.”

### 2.2. Tissue Microarray Array Construction

Three hundred and eight samples from Nigerian cohort were constructed as tissue microarrays (TMA) as previously described [30]. Breast tumour cores were taken from each FFPE donor tissue block that has been marked for the most representative points of tumour (both peripherally and centrally). A precision instrument (ALPHELYS MiniCore) was used to take representative cores of tissue (0.6 mm diameter, 3 mm height) from each sample, which was then arrayed into a recipient paraffin block in 11 × 15 core format. 

#### 2.2.1. Immunohistochemistry Method

**Figure 1:**
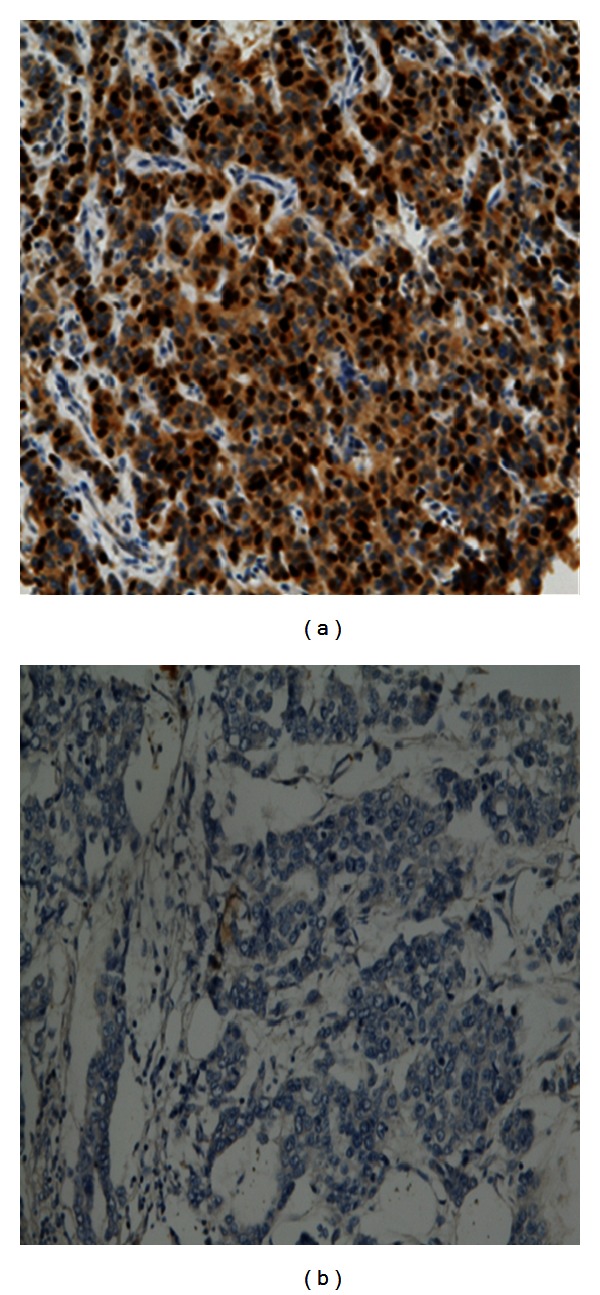
(a) and (b) show positive and negative immunoreactivity of KI-67 in Nigerian breast cancer magnification ×20.

 All the biomarkers required antigen retrieval except HER-2 and EGFR. Antigen retrieval was performed by microwaving the slides at 800 W for 10 minutes followed by 560 W for 10 minutes in citrate buffer (1 M sodium citrate at pH of 6.0) followed by cooling in running water immediately. The primary antibody for each biomarker (Table 1) was incubated for 60 minutes at room temperature. Diaminobenzidine tetrahydrochloride (DAB) solution was incubated for 10 minutes after which copper-sulphate solution (0.5% copper sulphate in 0.8% sodium chloride) were applied to the slides and incubated for 10 minutes each and counter stained with haematoxylin for 2-3 minutes, followed by rinsing in tap water. Slides were de-hydrated by immersing in three alcohol baths for 10 seconds and cleared in two xylene baths followed by application of cover slip. Negative and positive controls were performed by omitting the primary antibody and including control tissues as specified by the antibody supplier, respectively. Immunoreactivity expression of the biomarker in the nucleus was considered positive (Figure 1).

#### 2.2.2. Immunohistochemical Scoring

The scoring was performed using the modified histochemical score (H-score), a semiquantitative assessment. Staining intensity was scored from 0, 1, and 2 to 3, and the percentage of positive cells was determined for each score to produce a final score in the range 0–300. The cases were scored without knowledge of the patient outcome. The TMA samples were scored twice by one observer (JA). The mean of the scores were calculated to reach a final score. A proportion of these were counter scored by an observer (AG) to ensure reproducibility.

Table 1 shows the cut-off points used for the biomarkers analysis [31, 32]. For c-erbB2, the American Society of Clinical Oncology/College of American Pathologists Guideline Recommendations for Human Epidermal Growth Factor Receptor 2 Testing in Breast Cancer was used for assessment [33]. Equivocal (2+) cases were confirmed by CISH as previously described [34].

For molecular classification, Nielsen's method [35] was used. This comprises of Luminal A (ER, PR positive, and HER 2 negative), Luminal B (ER, PR HER 2 positive), basal (ER, PR, HER-2 negative, and CK5/6 and, or EGFR positive), HER2 (ER negative and HER2 positive), and an unclassified group (ER, PR, HER2 CK5/6, and EGFR negative).

### 2.3. Statistical Analysis

Statistical analysis was performed using SPSS 16.0 statistical software. Chi-squared analyses were used for interrelationships between the Nigerian and UK series and for comparison with clinicopathological parameters. The Kaplan–Meier survival method and the log-rank test were used for survival curves. Multivariate analyses using Cox proportional hazard regression models were performed, and from the model both the risk factor and 95% confidence intervals were generated. A two-sided *P* value of <0.05 was considered significant.

## 3. Results

The relationship between KI-67 expression in grade-matched breast cancer between Nigerian and UK women is summarised in Table 2. A significantly large proportion of breast tumours from Nigerian women showed high KI-67 expression compared with UK women, which accounted for 82.6% and 66.7%, respectively (*P* < 0.001).

In those tumours showing positive KI-67, a significant proportion of breast cancers from the Nigerian series was from patients that were premenopausal (*P* < 0.001) and diagnosed before 50 years (*P* < 0.001). Also, the tumours were significantly larger in size (*P* < 0.01), ductal carcinoma histological type (*P* < 0.001) with evidence of metastasis into lymph node (*P* < 0.001) and vascular invasion (*P* < 0.001) compared with the UK series (Table 3).

In the Nigerian series, positive expression of KI-67 biomarker was significantly correlated with negative expression of the steroid hormone receptors (ER and PgR), p21, p27, E-cadherin, BRCA-1, and Bcl-2 (all *P* < 0.001). In addition, a significantly larger proportion of Nigerian tumours that showed a positive KI-67 expression had positive relationship with EGFR (*P* = 0.003), p53, basal cytokeratins: CK56, CK14, triple negative, and basal phenotype using Nielsen's classification (all *P* < 0.001) compared to UK women. There was no positive correlation between the series in relation to HER-2 and P-cadherin (Table 4).

**Figure 2:**
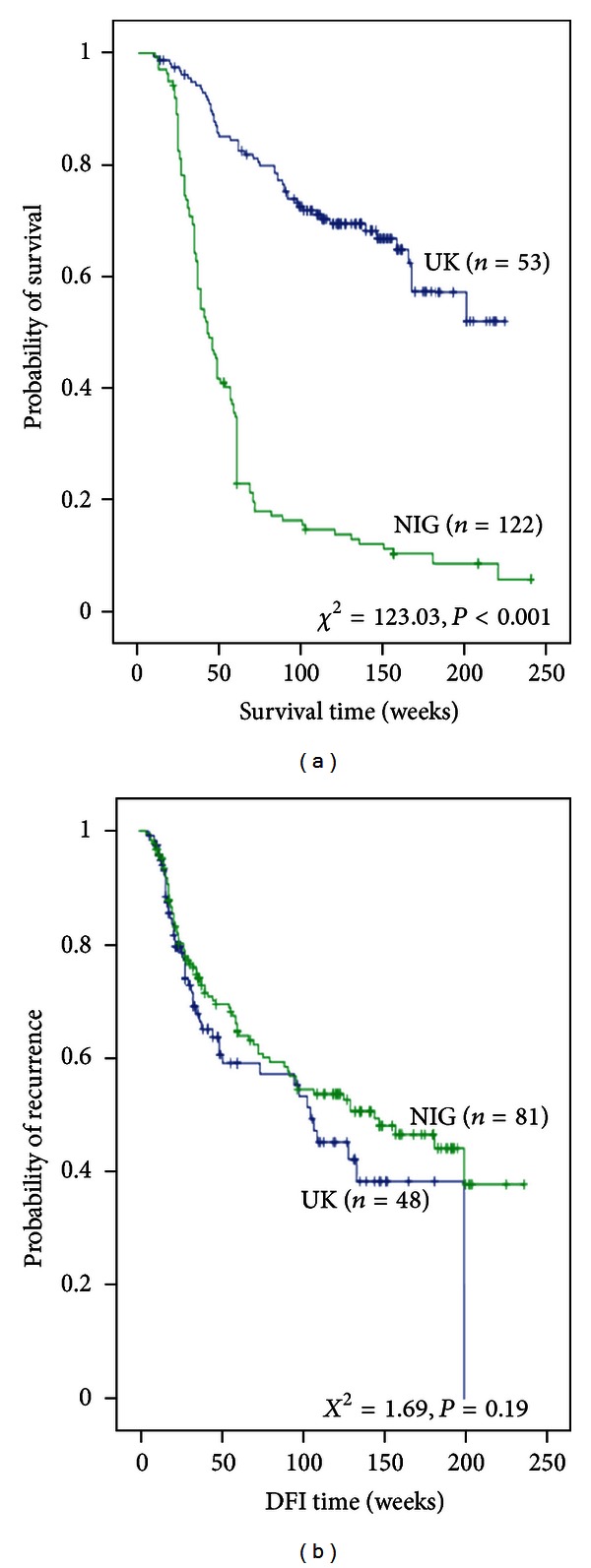
(a) and (b) show KI-67 positive expression in relation to BCSS and DFI between UK and Nigerian series.

Survival analyses were performed comparing the Nigerian and UK series in relation to both DFI and BCSS in the tumour expressing KI-67 biomarker. Nigerian women were significantly associated with BCSS (*P* = 0.001), but no significant association with DFI was observed (Figure 2). Cox multivariate analyses showed that race was not only associated with OS, but also its predictive power was independent of tumour size, lymph node status, and ER status (Table 5).

## 4. Discussion

KI-67 has been described as one of the important regulators of cell cycle for the maintenance of chromosomal integrity, and therefore any abnormalities in this cell proliferation marker expression might also contribute into passing deformed genes or chromosomes into daughter cells which may serve as impetus for carcinogenesis development with severe implications on tumour behaviour [36–38]. The prognostic significance of KI-67 is currently undergoing intense debate, in view of their importance in the development, proliferation, cell migration, and clinical outcome [39–41]. Also, up until now, there is no consensus regarding the significance of this marker in BC among different ethnicities.

In this study, protein expression of the KI-67 in 308 breast cancer cases from Nigerian women was evaluated and compared with histological tumour grade matched British women BC. Currently, there is a paucity of information on the protein expression of KI-67 in Nigerian breast cancer, and the results presented in this study showed that the tumour specimens obtained from Nigerian compared with British women were more likely to express KI-67. This is in support of other data on KI-67 protein expression at diagnosis in black women [39–41]. In ethnicity studies, KI-67 expression was higher in breast cancers from African-American compared with Caucasian women [21]. In addition high level of flow cytometric S phase measurement and mitotic count was also reported in African-American [23, 42, 43]. Previous studies on KI-67 expression in Nigerian and Saudi Arabian women also showed that KI-67 was overexpressed [39–41]. In addition, a strong relationship was established between poor prognostic indicators and KI-67 expression in this study, where Nigerian women with KI-67 expression were associated with patient diagnosed earlier in life, premenopausal, larger tumour size, lymph node involvement, vascular invasion, exhibited a basal phenotype, and triple negative, lacked hormone receptors, BRCA1, and shorter BCSS compared with British women. This is in line with previous studies on Caucasian women, where aggressive tumours are associated with KI-67 expression [8, 16, 44, 45]. Similar aggressive features observed in this study were also reported on high KI-67 expression in African-American compared with Caucasian women [20, 21]. In Nigerian series, the high expression of this proliferative marker might have contributed to their aggressiveness, having been previously associated with aneuploidy that are responsible for high cell division turnover rate, which has been linked with aggressive tumours [11, 12]. It may also be involved in the metastatic potential of this BC, because of its association with majority of tumour that had already invaded both the lymphatic and blood vessels at diagnosis. In addition, this marker may also be involved in the lack of response to endocrine therapy and conventional chemotherapy in Nigerian tumours. KI-67 overexpression would have also contributed to high mortality rate observed in Nigerian compared to UK women as a result of its relationship with basal phenotype, lack of hormonal receptors and BRCA1. Low KI-67 expression and high hormone receptors expression are found to be associated with improved endocrine therapy [46], and only few basal phenotype and BRCA1 are likely to response to chemotherapy [47]. Furthermore, KI-67 has also been reported to be a predictor of poor outcome in aggressive BC [45, 48], in line with this, Nigerian BC had a shorter BCSS compared with British women. Race was observed to be independent prognostic factor in the tumours expressing KI-67, and therefore this marker might have also contributed to the difference in the tumour biology observed between the black and Caucasian women, and also using KI-67 expression to stratify black women with BC may improve the prognostic significance of clinical response to treatment.

In conclusion, KI-67 expression was found to have contributed to the difference in the tumour biology and poor overall survival in Nigerian BC women compared with British grade counterpart. Therefore, targeting this marker in the indigenous black women with BC might reduce the dismay outcome in the black women with BC.

## Figures and Tables

**Table 1:** Sources, dilution, distribution, cut-offs point, and pretreatment used for revalidation.

Antibody	Clone	Source	Dilution	Distribution	Scoring System	Cut-offs	Pretreatment	Positive control	Negative control
Bcl-2	124	Dako-Cytomation	1 : 100	Cytoplasm	% of positive cells	>10% (positive)	Antigen retrieval microwave	Normal breast acini	Omitting the antibody
BRCA1	Ab-1 (MS110)	Calbiochem	1 : 150	Nuclear	% of positive cells	<25% (negative)	Antigen retrieval microwave	MCF 7 cells	Omitting the antibody
Ck5/6	M7237	Dako-Cytomation	1 : 60	Cytoplasm	% of positive cells	≥10% (positive)	Antigen retrieval microwave	Known case of CK56 BC	Omitting the antibody
E-cadherin	NCH-38	Dako-Cytomation	1 : 100	Cytoplasm and membrane	% of positive cells	≥100 H score (positive)	Antigen retrieval microwave	Normal gastric mucosa	Omitting the antibody
EGFR	31G7	Novocastra	1 : 30	Membrane	% of positive cells	≥10% (positive)	Not required	Myoepithelial cells of normal duct in normal mammary gland	Omitting the antibody
erbB2	Polyclonal	Dako-Cytomation	1 : 100	Membrane	Table 2.1	Table 2.1	Not required	Known case of erbB2 strong BC expression	Omitting the antibody
ER	1D5	Dako-Cytomation	1 : 200	Nuclear	% of positive cells	≥0 (positive)	Antigen retrieval microwave	Normal breast acini	Omitting the antibody
Ki-67	MIB1	Dako-Cytomation	1 : 25	Nuclear	% of positive cells	<10% (low)	Antigen retrieval microwave	Human tonsil tissue	Omitting the antibody
P-cadherin	NCL-P-cad	Novocastra	1 : 200	Cytoplasm	% of positive cells	≥5% (positive)	Antigen retrieval microwave	Known case of P-cadherin strong BC expression	Omitting the antibody
PgR	PgR	Dako-Cytomation	1 : 150	Nuclear	% of positive cells	≥0 (positive)	Antigen retrieval microwave	Normal breast acini	Omitting the antibody
p21	EA10	Abcam	1 : 25	Nuclear	% of positive cells	≥10% (positive)	Antigen retrieval microwave	Normal breast acini	Omitting the antibody
p53	DO7	Novocastra	1 : 50	Nuclear	% of positive cells	>10% (negative)	Antigen retrieval microwave	Normal breast acini	Omitting the antibody

**Table 2:** Relationship between KI-67 marker expression in Nigeria and UK series.

KI-67	Nigeria (%)	UK (%)	*χ* ^ 2^ value	*P* value
Negative	46 (17.4)	82 (33.3)	**85.40**	**<0.001**
Positive	218 (82.6)	164 (66.7)		

**Table 3:** Relationship between clinicopathological parameters in Nigerian and UK tumours expressing KI-67.

Variables	KI-67 positive expression			
	Nigeria (%)	UK (%)	*χ* ^ 2^ value	*P* value
Age (years)				
≤50	136 (62.4)	62 (37.8)	**22.65**	**<0.001**
>50	82 (37.6)	102 (62.2)		
Menopausal				
Pre	157 (72.0)	60 (36.8)	**47.15**	**<0.001**
Post	61 (28.0)	103 (63.2)		
Sizes (cm)				
≤2	18 (8.3)	73 (44.5)	**67.79**	**<0.001**
>2	200 (91.7)	91 (55.5)		
Lymph node involvement				
Negative	16 (7.3)	97 (59.1)	**120.58**	**<0.001**
Positive	202 (92.7)	67 (40.9)		
Vascular invasion				
Negative	51 (23.4)	86 (52.4)	**34.32**	**<0.001**
Positive	167 (76.6)	78 (47.6)		
Tumour type				
Typical medullary	3 (1.4)	1 (0.6)	**43.10**	**<0.001**
Atypical medullary	4 (1.8)	7 (4.3)		
Tubular	1 (0.5)	0 (0.0)		
Lobular	3 (1.4)	8 (4.9)		
Ductal NST	189 (86.7)	105 (64.8)		
Mucinous	4 (1.8)	0 (0.0)		
Tubulolobular	0 (0.0)	7 (4.3)		
Mixed NST	14 (6.4)	30 (18.5)		
Others	0 (0.0)	2 (1.2)		

NST: no special type. Others: metaplastic, spindle, and alveolar lobular histological type.

**Table 4:** Relationship between biomarker expression in UK and Nigerian tumours expressing KI-67.

Variables		KI-67 positive expression		
	Nigeria (%)	UK (%)	*χ* ^ 2^ value	*P* value
BRCA1				
Negative	143 (80.8)	31 (21.5)	**112.34**	**<0.001**
Positive	34 (19.2)	113 (78.5)		
Bcl-2				
Negative	101 (57.4)	53 (37.6)	**13.15**	**<0.001**
Positive	75 (42.6)	88 (62.4)		
CK5/6				
Negative	108 (58.7)	137 (84.6)	**27.90**	**<0.001**
Positive	76 (41.3)	25 (15.4)		
CK14				
Negative	97 (58.8)	148 (90.8)	**44.5**	**<0.001**
Positive	68 (41.2)	15 (9.2)		
ER				
Negative	169 (84.5)	45 (28.0)	**118.17**	**<0.001**
Positive	31 (15.5)	116 (72.0)		
EGFR				
Negative	119 (66.5)	123 (80.4)	8.08	**0.003**
Positive	60 (33.5)	30 (19.6)		
E-cadherin				
Negative	119 (73.5)	65 (40.1)	**36.7**	**<0.001**
Positive	43 (26.5)	97 (59.9)		
HER-2				
Negative	148 (81.8)	140 (87.5)	2.13	0.14
Positive	33 (18.2)	20 (12.5)		
PgR				
Negative	126 (79.7)	68 (41.7)	**48.53**	**<0.001**
Positive	32 (20.3)	95 (58.3)		
p27				
Negative	131 (68.9)	45 (46.4)	**13.77**	**<0.001**
Positive	59 (31.1)	52 (53.6)		
p21				
Negative	152 (83.1)	65 (61.9)	**16.07**	**<0.001**
Positive	31 (16.9)	40 (38.1)		
p53				
Negative	1 (1.0)	10 (14.7)	**13.11**	**<0.001**
Positive	104 (99.0)	58 (85.3)		
P-cadherin				
Negative	81 (41.5)	66 (44.9)	0.38	0.54
Positive	114 (58.5)	81 (55.1)		
Triple negative				
No	68 (45.6)	128 (79.0)	**37.10**	**<0.001**
Yes	81 (54.4)	34 (21.0)		
Classification				
Basal	56 (49.1)	15 (10.2)	**149.02**	**<0.001**
HER-2	26 (22.8)	9 (6.2)		
Luminal A	27 (23.7)	113 (77.4)		
Luminal B	5 (4.4)	9 (6.2)		

**Table 5:** Cox multivariate analysis of probability of survival in tumours expressing KI-67 in Nigerian and UK breast cancer.

	KI-67 positive expression			
Variables	*P* value	Hazard ratio	95% CI	
			Lower	Upper
Racial difference Nigeria versus UK	<0.001	4.18	2.68	6.53
Lymph node	0.69	1.09	0.71	1.67
Tumour size	0.12	1.12	0.96	1.31
ER	0.21	0.79	0.55	1.14
